# Preparing for Death While Investing in Life: A Narrative Inquiry and Case Report of Home-Based Paediatric Palliative, End-of-Life, and After-Death Care

**DOI:** 10.3390/children10111777

**Published:** 2023-11-02

**Authors:** Michelle Noyes, Angela Delaney, Meagan Lang, Mellissa Maybury, Susan Moloney, Natalie Bradford

**Affiliations:** 1 Department of Paediatrics, Gold Coast University Hospital, Southport, QLD 4215, Australia; michelle.noyes@health.qld.gov.au (M.N.); meagan.lang@health.qld.gov.au (M.L.); susan.moloney@health.qld.gov.au (S.M.); 2 Paediatric Palliative Care Service, Queensland Children’s Hospital, South Brisbane, QLD 4101, Australia; angela.delaney@health.qld.gov.au; 3 Centre for Children’s Health Research, Children’s Health Queensland Hospital and Health Service, South Brisbane, QLD 4101, Australia; 4 Queensland Children’s Tumour Bank, Child Health Research Centre, University of Queensland, South Brisbane, QLD 4101, Australia; mellissa.maybury@uq.edu.au; 5 School of Medicine, Griffith University, Southport, QLD 4215, Australia; 6 Cancer and Palliative Care Outcomes Centre, Queensland University of Technology, Brisbane, QLD 4001, Australia

**Keywords:** palliative care, paediatric, home care services, communication, qualitative research, interprofessional relations, symptom management

## Abstract

Paediatric palliative care is pivotal for addressing the complex needs of children with incurable diseases and their families. While home-based care offers a familiar and supportive environment, delivering comprehensive services in this context is challenging. The existing literature on home-based palliative care lacks detailed guidance for its organization and implementation. This qualitative narrative inquiry explores the organization and provision of home-based paediatric palliative care. Data were collected from healthcare practitioners using conversations, storytelling, and reflective journaling. Schwind’s Narrative Reflective Process was applied to synthesize the data, resulting in an in-depth case description. The narrative approach illuminates the complexities of home-based paediatric palliative, end-of-life, and after-death care. Key findings encompass the importance of early-care coordination, interprofessional collaboration, effective symptom management, emotional and psychosocial support, and comprehensive end-of-life planning. Through the case study of the child patient, the challenges and strategies for providing holistic, family-centred care within the home environment are described. Practical insights gained from this report can inform the development and improvement of home-based palliative care programs, benefiting researchers, practitioners, and policymakers seeking to optimize care for children and families in similar contexts.

## 1. Introduction

Despite significant advancements in cancer treatment for children, a significant proportion (15–20% in developed nations) will die from the disease [1]. Children can also be diagnosed with non-malignant life-limiting conditions that shorten their lifespan [2]. In such circumstances—and regardless of the economic status of a nation—paediatric palliative care offers a myriad of supportive interventions to address the physical, emotional, spiritual and social concerns of both the child and their family [3]. Consequently, paediatric palliative care has gained recognition as an integral sub-specialty that plays a pivotal role in optimising outcomes for children and their families when faced with incurable disease [4,5].

Recognising there is no universal right or wrong place for palliative and end-of-life care, many families, where possible, choose to receive such care for their child in the comfort of their own home [6]. The home environment offers not only a sense of security and normalcy but also serves as a place of refuge, providing a level of comfort to both the child and family that is challenging to re-create elsewhere [7]. This decision remains deeply personal and may be made to respect the child’s wishes or maintain the family’s role as the primary caregiver in a familiar environment [8].

However, the decision for home care introduces complex challenges; children nearing the end of life experience a high burden of symptoms including pain, fatigue, dyspnoea, irritability, anxiety and fear [9]. Parents and siblings face the devastating prospect of losing their loved one, resulting in anxiety, fear and grief, and home care requires they also remain capable of managing difficult symptoms when navigating their child’s end of life [4]. Managing these factors and addressing the resulting distress on both the child and the family are crucial aspects of holistic paediatric palliative care [3,10]. Hospitals and hospices offer access to dedicated staff and resources to manage and navigate the unpredictable path of a child’s dying. Achieving the same range of supports in the home environment poses challenges on many levels [11]. The variations in service components and multiple service providers, at a highly emotional time and in the home environment, is a complex intervention [12].

Despite the existing literature describing the benefits of home-based paediatric palliative care, examples of how to organise such complex care remain limited [6]. Specifically, there is a lack of comprehensive guidance or detailed descriptions of practical strategies for organizing and delivering high-quality home-based paediatric palliative care. This highlights the need for in-depth exploration and documentation of the processes, challenges, and best practices involved in establishing and managing home-based paediatric palliative care programs.

The aim of this paper was to employ these methods to describe the provision of home-based paediatric palliative care and contribute knowledge and guidance for practitioners to improve the experiences of children and families facing end of life.

## 2. Methods

### 2.1. Study Design

We used qualitative narrative inquiry to present a case report of a single child patient, with a focus on capturing events over time. Narrative inquiry and case report are methodologies of learning about complex phenomena based upon understanding obtained through rich description and analysis of how components work together [13]. These relational methodologies explore complex real-life experiences, making meaning and amplifying voices and perspectives to provide an in-depth understanding of a given sociocultural context [14]. The nuanced and personal narrative offers an authentic source of knowledge, exploring complex phenomena [15]. Focusing on one case study allows for a detailed exploration of the specific context and unique dynamics of the care provided [16]. The philosophical underpinnings of the approach theorize that experience is relational, temporal and situational—and, when intentionally explored over time, can be educational [17]. The process of engaging in thoughtful reflection of experiences can make meaning of them and expand ways of being (ontology), knowing (epistemology) and doing (praxis) [18]. The knowledge gained can thus offer understanding and insight that may be applicable to the reader’s own context.

### 2.2. Data Collection and Analysis

The case was chosen as it provided an example of home-based care involving multiple professionals. Data were collected from four healthcare professionals involved in provision of home care to the case under study. The professionals were asked to consider how they provided home-based care to the child and family. Initially, conversations between health professionals were used to reflect upon their individual involvement in the case, and the impact upon their professional lives. Storytelling was then used to further develop the case study, moving away from individual experiences to collectively describe what was done, when and why. Finally, reflective journaling was used to obtain descriptions of the processes and purposes of care from the professionals, providing multiple perspectives [19]. Each professional was asked to document their experiences of providing care, again focusing on what was done, when and why. Prompts were used to elicit information to further reflect upon meanings considering the social, temporal and spatial dimensions of inquiry [19]. These data were shared between the group, prompting further discussion and reflection of care provision. Data were synthesised using principles from Schwind’s Narrative Reflective Process [20] to provide a rich description of the case, allowing the tacit knowing and fragments of experience to be described and made sense of [19]. This process is grounded in the understanding that personal experiences and stories are powerful tools for shaping perceptions and experiences [18]. The synthesized data were then developed into a temporal narrative. To facilitate a credible narrative, this was member-checked, maintaining audit trails. This process facilitated exploration of personal experiences of providing care in a reflective manner with an emphasis on the interactions between all involved in home care. The findings are presented as a storytelling of the case under study informed by principles of paediatric palliative care [21]. Our research protocol and methods were reviewed and endorsed by the local Human Research Ethics Committee. In line with ethical standards, we obtained written parental consent for publication of this case study; the parents also reviewed the manuscript and requested inclusion of the child’s name and details in the publication in honour of their son’s life. While we followed the standards for obtaining informed consent [22], it is worth noting that the issues discussed below describe the organization and processes of home care. In this sense, the case study is also about the people involved in providing and receiving care.

## 3. Case Presentation

Riordan was a determined 10-year-old boy with big dreams. He was excelling in soccer and athletics, participating in state-level competitions. He was also a bright and dedicated student and school leader, winning school awards for academic excellence, particularly in science, mathematics, and technology. He lived with his parents and 12-year-old brother, with whom he shared a very close bond.

### 3.1. Diagnosis and Home Care Decision

After a few short weeks of symptoms, investigated with an MRI scan and surgical biopsy, Riordan was diagnosed with diffuse intrinsic pontine glioma (DIPG), an aggressive form of childhood brain cancer with minimal treatment options and an abysmal prognosis. His parents were informed of the disease trajectory at the time of diagnosis and elected to shield Riordan and his sibling from the prognosis for as long as possible.

Choosing to provide care at home was a decision made early in the family’s journey. Six weeks of radiation treatment were completed as day trips from the family home, with the family preferring to commute 100 km every day instead of staying in hotel accommodation near the radiation centre. Riordan’s parents believed he was happiest at home with his beloved dog and older brother for company and entertainment. This decision was made easier by the fact the family lived just 15 min from their local regional hospital, making it convenient to attend local hospital reviews, and enabling local hospital staff to provide consultations at home.

### 3.2. Palliative Care Referral and Role of the Care Coordinator

A few months following diagnosis and radiation treatment, Riordan experienced an increase in clinical symptoms. His parents requested an early referral to the Paediatric Palliative Care Service (PPCS), to assist with navigating the difficult road ahead. In Queensland, Australia, when families live far away from the tertiary children’s hospital oncology service and the specialist PPCS, the role of the care coordinator is often delegated to a specialist paediatric nurse at the local regional hospital. In Riordan’s case the paediatric oncology/palliative care nurse practitioner (NP) at the local regional hospital took on the role as care coordinator for Riordan and his family.

It is important for the care coordinator to be introduced to the family shortly after diagnosis to ensure there is a point of contact for the family and sufficient time to build a therapeutic trusting relationship. It is extremely beneficial for the care coordinator to have knowledge of the anticipated disease trajectory to assist in preparing parents to plan for goals of care and to anticipate symptoms before they arise.

In the early stage of palliative care, the local care coordinator phoned the parents every one to two weeks and facilitated referrals to the local regional hospital paediatrician and other members of the interprofessional team to assist with providing care close to home. This included referrals to physiotherapy, occupational therapy, speech therapy, social work and psychology. The local team supported applications for financial assistance through government and non-governmental agencies to boost reduced family income while parents took leave from employment to care for Riordan. Applications were also made for funded practical assistance to pay for home cleaning and garden maintenance to give the parents time with their boys.

Monthly interprofessional case-conference team meetings provided updates for local health professionals on Riordan’s progress and allowed for timely intervention from specific team members when needed. Riordan was supported to return to school and participate in sporting activities for as long as possible.

The interprofessional meetings also provided staff with opportunities for peer support through sharing of approaches and strategies to manage challenges. While there were no formal meetings between the regional centre, the PPCS and the tertiary oncology service, team members were available to support the regional centre as required. This support can be particularly helpful for the regional care coordinator, who often has the most regular contact with families and may be the team member who navigates difficult conversations around ceilings of care and advance care planning.

Without formal processes in place, communication between teams and services across different institutions and physical locations can be challenging. In Riordan’s case, the local care coordinator informed the understanding of disease progression and plans for management from conversations with the parents, phone calls and emails from the tertiary hospital and through filtering information from notes in shared electronic medical records.

### 3.3. Family-Centred Communication and Interprofessional Care

One year following diagnosis, Riordan had tumour progression with a significant decline in his physical function, speech, and an increase in symptoms. Riordan was now aged 11 and in his final year of primary school, and his brother was 13 and in the first year of high school. His brother expressed frustration about not doing fun activities anymore and always having to be at home. He was resenting the fact that activities were planned around what his brother wanted to do. Riordan was reluctant to leave the home, due to the effort required and feeling self-conscious about changes to his physical appearance due to side effects of the steroid medication.

As Riordan’s condition continued to deteriorate, his mother requested guidance on how to talk to both boys about the disease progression and the reality that Riordan would soon die. Riordan’s mother shared concerns that the boy’s incomplete understanding of what to expect was impacting the family’s opportunities to connect around what was important to them. Riordan’s father was hoping to protect the boys from the sad reality that Riordan would die, and was initially hesitant to engage in these conversations.

Riordan was referred to the regional hospital paediatric clinical psychologist to provide an opportunity for him to express his fears, worries and sadness. During the initial assessment, Riordan acknowledged the possibility he may die. However, Riordan’s primary concerns related to feelings of reduced connection with his brother, fears of falling behind at school, not meeting his potential, and a fear of being alone in his wheelchair in the future, questioning who would care for him when his parents grew old. He also wondered if he should wait to feel better before planning for his starlight wish (a charity support program that grants wishes to children with serious illness) or whether to consider taking it soon. This raised concerns about Riordan’s understanding of the trajectory of his illness. Riordan was supported to share these fears with his mother and the care coordinator. All these conversations occurred when Riordan’s speech was rapidly declining; however, throughout his illness Riordan’s cognition never faltered, and he continued to find ways to communicate his needs.

The strained family relationships and decline in Riordan’s verbal communication motivated both his parents to progress with difficult discussions about dying, so Riordan would not have to deal with his fears of an uncertain future alone and the family could connect through a shared understanding. In such circumstances, it is important to recognise that although the child and their sibling may recognise death is a possibility, it should not be assumed they understand this is the likely or inevitable outcome. It is also important to recognise that although we may shield them from this information, we cannot protect them from their fears or the burden they may feel in managing these fears alone.

Planning for how to initiate these tender conversations and sharing ideas and strategies on how to introduce the topic was the key to empowering Riordan’s parents. The care coordinator developed an understanding of the family’s previous experience of grief and loss and identified the language Riordan’s parents used for this. Mirroring the family’s language and the words they use is recommended practice to enhance shared understanding when having difficult conversations [23].

The parents wished to speak with the boys separately, to ensure they could provide support appropriate for each child. Planning for this involved how to bring up the conversation and who would be best to talk with each child. A trusted and experienced health practitioner was offered to support this, but the parents felt the conversations they had with the nurse practitioner prepared them to talk to the boys alone.

Riordan’s parents shared that having this conversation with the boys was the hardest task they had ever done. They were initially overwhelmed witnessing their sons’ immense sadness, distress, and shock on being told Riordan would soon die. However, they reflected that this difficult, honest conversation with the boys changed the way everyone in the family communicated with one another. Their grief was now shared and their goals of what was important for each other was also acknowledged. The elephant in the room was no longer there.

Riordan’s goals included wanting to graduate from primary school, watch his brother play soccer more, and spend time with friends and those close to him. Together, the health practitioners and staff from Riordan’s school worked collaboratively with Riordan and his family to achieve his wishes. Riordan’s mother helped him prepare a graduation speech and the ceremony was brought forward to ensure Riordan was well enough to attend. Achieving this goal gave Riordan enormous pride and inner peace. The school gifted Riordan a beautiful book of cards from his classmates for his 12th birthday, sharing with him their best wishes, favourite memories, and the personal stories of the impact he had on them throughout their time at primary school.

For Riordan’s brother, knowing the time they had together was going to be very short, he chose to be much more involved in Riordan’s care, helping Riordan at mealtimes, assisting their father with Riordan’s daily showers, and helping to operate the hoist to move Riordan from room to room. The brothers shared more time together, playing with their precious dog, watching TV, and laughing together over old family videos. Riordan’s brother became an enthusiastic contributor during home reviews, sharing stories and laughter with Riordan ’s every cue.

### 3.4. Hospice and Community-Nursing Referrals

Hummingbird House, a dedicated stand-alone children’s hospice, was introduced early to the family as an option for short breaks, symptom management, and a home-like environment for end-of-life and after-death care. The hospice was a ninety-minute drive from the family home and only 20 min drive from the tertiary children’s hospital and radiation centre. A second course of palliative radiotherapy was offered at time of disease progression, and the family elected to stay at Hummingbird House during re-irradiation instead of travelling the longer distance from home; this also coincided with the timing of bathroom renovations in the family home. The renovations were necessary to accommodate Riordan’s tilt-in-space shower chair, to enable him to have the daily showers that he enjoyed.

Additional items of equipment were needed to optimise Riordan’s quality of life at home. Collaboration with the specialist adult palliative care service at the regional hospital, and in particular the occupational therapist (OT), helped access equipment in a timely manner. The OT assisted with loan of a motorised hospital bed, tilt-in-space wheelchair, shower chair, recliner chair, hoist, and portable suction unit.

Riordan experienced increased difficulty with swallowing, and subsequently with managing oral secretions and taking oral medications. He did not consent to a nasogastric tube for medications and, due to increasing pain, transition to delivery of medications by a subcutaneous route was considered the appropriate option. At this time, a community nursing agency was introduced to the family to provide additional support in the home with daily visits by a palliative care nurse to prepare and reload the subcutaneous infusion and ensure adequate rescue doses of medication were prepared for the parents to administer.

While the community nurses were experienced in palliative care, they had minimal experience in caring for dying children, and were supported by the care coordinator and the PPCS after-hours telephone service on weekends.

### 3.5. Symptom Management and Pain Control

Administration of medications and therapies at home requires meticulous planning, and the use of a written Symptom Management Plan (SMP) is a crucial tool to support symptom control [24]. It is important to plan ahead and have a starter pack of parenteral medications, an ambulatory infusion pump, and associated consumables available in the home to enable easy transition to subcutaneous delivery of medications when required. Monitoring of medication supply and consumable stock for infusions is essential, particularly in locations where medications may not be readily available at the hospital or community pharmacy.

The advanced-practice clinical role of a nurse practitioner partners well with the specialty of paediatric palliative care to enhance the delivery of coordinated and holistic care to the child and family. A nurse practitioner can provide comprehensive and focused assessments, plan care in collaboration with numerous teams, and prescribe pharmacological and non-pharmacological interventions within the scope of clinical practice. A nurse practitioner with experience and expertise in paediatric palliative care can also prepare families for what to expect towards the end of life and plan care according to how families wish their child to be cared for.

It was important for Riordan’s family to have prior knowledge of possible medical events that could occur as the disease progressed; this included potential for seizures, increasing pain, dyspnoea, worsening dysphasia and decrease in level of consciousness. Along with knowing about possible acute medical events, conversations around the level of medical intervention the parents wanted to be provided to Riordan were essential. While Riordan and his brother were included in many conversations about his care, there were some conversations his parents wished to have separately. It can be hard to find privacy to have conversations about advance care planning in a home setting, and at times these conversations were had in the front yard of the family home where parents could talk openly. Providing opportunities for Riordan’s parents to meet with the paediatrician at the hospital also facilitated formal advance-care-planning discussions. A Paediatric Acute Resuscitation Plan (PARP) was documented by Riordan’s paediatrician to ensure that in the event that the ambulance service was called to the home or Riordan was admitted to hospital, the medical care focussed on treating pain, agitation and other distressing symptoms, rather than sustaining life.

As well as home visits through community nursing agencies, regular review from paediatric and palliative services are important aspects to achieve optimal symptom management and pain control [25]. The use of telemedicine and remote consultations are beneficial in achieving this [26]. Regular phone contact with the specialist PPCS guided and supported the care coordinator and community nurses with titration of infusion doses to manage pain, agitation, and dyspnoea. The family also contacted the PPCS for after-hours support on a toll-free number to manage a situation where Riordan experienced escalating pain and respiratory distress towards the end of his life.

### 3.6. Emotional and Psychosocial Support

Key health professionals continued to support the family throughout this time, with the capacity to lean into the families’ experiences as they presented. Music therapy and psychology were able to support the strengths and bear witness to love within this family. The brothers had previously connected through playing and ‘doing’, and needed to find new ways of connecting. Psychology and music-therapy sessions occurred weekly and included opportunities for meaningful contribution, connection, and memory making.

Music therapy can offer potential for change and connection in deep and meaningful ways where words are insufficient. Elements of music (rhythm, melody, harmony, dynamics, timbre, form) address our basic human sensory needs, providing a bridge to safely communicate and externalise thoughts and feelings [27].

Music therapy for Riordan and family was particularly helpful during his final months. The family embraced the opportunity of ‘doing’ together with much enthusiasm, energy and laughter and tears, while Riordan was very much a vocal contributor, despite losing his capacity for spoken words. The family unit engaged in all music therapy sessions, initially fortnightly then weekly, as part of their ritual of the things they could engage in together. During these sessions Riordan and his family were able to express their experiences through supported music expression in order to make meaning. Riordan’s family used robust rhythmic interventions, song singing, sharing, and song writing to express what they needed, individually, while the other family members and the music therapist held the space. Riordan dictated how the session would unfold, embracing the predictability and structure music affords amidst the unpredictability, while the family remained connected to each other and the music.

During individual and family psychology reviews, Riordan was supported to explore feelings of frustration, grief and loss related to changes in function, reduced opportunities for meaningful participation, and dreams not realised. Riordan was also supported to ‘make space’ for fears of the future, including fears of dying, when he identified the fact that pushing these fears away was no longer effective. The psychologist facilitated family discussions around their shared struggle and suffering, to support opportunities for increased family connection and reduce feelings of isolation. Riordan was supported to explore what he wanted his family and others to know as he approached the end of his life.

Riordan’s determination to communicate was matched by the determination of his family to understand him. His iPad was initially used to assist communication; however, its utility was impacted as his fine motor control declined. The family interpreted Riordan’s speech when difficult to understand, and eventually assisted him to spell out words and explore related themes, as guided by his cues. Acceptance and Commitment Therapy (ACT) value and communication cards were also a powerful tool to allow Riordan to set the agenda and identify themes that were important to him.

Supported by this interprofessional approach, the family developed a shared narrative of Riordan having lived a full and meaningful life, although his life was short. The family were able to openly share their grief, disappointments, sadness, love, spiritual beliefs and wishes with each other. The narrative of this families’ engagement with health professionals illustrates how families are often in the paradox of preparing for death while investing in life.

### 3.7. End-of-Life and After-Death Care

The planning for Riordan’s death, and for how he would be cared for after death was as important as caring for him in life. Families remember this time vividly, and the care provided will be remembered for their lifetime. The team, therefore, aims for families to remember this as a peaceful and sacred time.

To plan and prepare for these moments, sensitively asking parents and siblings about how much they want to know and how much they want to be included can guide conversations. Anticipation of symptoms and acute events is crucial to ensure both the comfort of the child and to also mitigate the distress of the family. It is important these conversations are initiated as soon as possible when it becomes evident a child is entering the final phases of life. This ensures parents, siblings and other extended family members have time to ask questions important to them and time to prepare for the process of the child’s death, and allows time to say goodbyes.

Parents may feel comforted by the knowledge that there is the option to change the location of care to a hospital or children’s hospice if the child’s symptoms are not able to be managed adequately in the home. In such cases, it may be helpful to reframe expectations and acknowledge the extent of care that has already been provided in the home.

For children who do die at home, a detailed after-death care plan ensures the child’s body is cared for appropriately, including arrangements for the transfer of the child’s body to a funeral home at a suitable time for the family. Families may also wish to undertake cultural practices or rituals in caring for their child’s body. Understanding these and documenting them in the after-death care plan ensures these wishes are understood and respected. Memory-making at this time can offer a poignant and lasting impression which may help in processing grief and loss. Collaborations with support charities such as Precious Wings who provide memory boxes have been pivotal connections to support the broader education of health professionals as well as being a healing resource for families [28].

Other collaborations with industry have enabled families to provide after-death care to their child for a longer time in humid, hot climates such as Queensland. The use of portable frozen dry-ice products can be used in the home to help cool the child’s body after death. Some families may choose to care for their child’s body at home for several days, and health professionals can now support families with this process [29]. The integrity of the child’s body can be maintained with the rotation of dry-ice sheets to ensure the body is cooled to a similar temperature as it would be in a mortuary. Some families find comfort in being able to provide ongoing care to their child’s body, and this may include bathing, moisturising, painting nails, brushing hair, etc.

### 3.8. Post Mortem Tumour Donation

In Riordan’s case, his parents wished for his brain tumour to be donated to research and his corneas to transplant services after his death. As these wishes were made known, the interprofessional care team was able to plan for this and document the procedures in Riordan’s after-death care plan. Riordan was aware of his parent’s wishes to proceed with tissue donation following his death, and he respected their wishes and also asked not to be included in these conversations as this was upsetting for him. Families may choose to donate tissue or tumour post mortem for a number of reasons. This may include altruistic reasons, in the hope of advancing science and finding answers to why cancer occurred, or finding a treatment to help others, which can be a step forward in the healing process [30]. Families may also hope to find answers for themselves, for example to definitively know the type of cancer or to alleviate lingering concerns about possible treatment options [31]. Riordan’s parents also requested corneal donation in the hope that Riordan would continue to see the world through others.

The coordination of autopsy and tissue donation for research can be logistically challenging, needing to be completed in a timely manner to maintain tissue viability, while being sensitive to the family’s recent loss. While there is no data which accurately define the ideal post mortem interval (PMI)—the time of death through to final storage of tissue—it is generally accepted that post mortem tissue donations should be performed as ‘rapid autopsies’ within 8 h of death if viable tumour cells are to be obtained for live cell culture [32].

The coordination of staff to perform a rapid autopsy is highly dependent on the time of death and the release of the body to the mortuary. Unless there are multiple departmental staff members who are on-call and available at all times, the ideal situation for post mortem tumour donation is where a death occurs between the hours of 3 a.m. and 4 p.m. on a weekday, when staff are more likely to be available, and the tumour is extracted for downstream processing within 6 h of time of death. Depending on the size of the tumour and number of metastases, the processing of tissues for biobanking and research can take approximately 2–8 h. Where the PMI is less than 72 h, the tumour material is still considered highly valuable and can be utilised for genomics (e.g., DNA mutations) and proteomics (e.g., biomarkers) [33].

The post mortem donation of inoperable tumours such as those from DIPG is highly valuable to researchers, as diagnostic biopsies from living patients are often small core biopsies and the entire specimen is required for histopathology and clinical diagnosis. Post mortem donations provide researchers with adequate representative tissue to study the tumour’s pathophysiology, and facilitate the identification of biomarkers, tumour evolution processes, and therapeutic targets.

Riordan died peacefully in his bedroom surrounded by his parents and brother in the early evening of a Monday. His parents were equipped with the after-death care plan and contacted the PPCS after-hours service, who were able notify the interprofessional care team of Riordan’s death and activate the planned tissue donations. As part of Riordan’s after-death plan, his parents arranged for transfer of his body to the funeral home that evening. The funeral director transported Riordan’s body to the hospital mortuary on the Tuesday morning. The tumour biobanking coordinator and the eye bank coordinator were ready on site, and began the process of post mortem tissue donation, with prioritisation of the eye biobank for transplant services, and then the tumour donation for research purposes. The tumour donation was assisted by the highly trained mortuary staff and the anatomical pathologist, who notified the funeral director that Riordan’s body was ready for transport back into the funeral home once the donation had concluded. Tumour specimens were then transported back to a research laboratory, where the scientific staff worked extensive hours to process and de-identify hundreds of vials of snap-frozen and cryopreserved samples under aseptic conditions for future unspecified research. Riordan’s deidentified tumour tissue was also sent to external academic research laboratories which are led by internationally recognised DIPG scientific research experts.

The experience of facilitating post mortem tissue donations from children can be confronting for biobanking staff, and access to free professional counselling services are available to all of those who are involved. Staff will often conclude the tissue donation with an informal debrief, and have often remarked that although they are far removed from clinical care, they find the process of honouring the child’s legacy both rewarding and humbling.

### 3.9. Bereavement Care

Following Riordan’s death, bereavement care included phone calls to the family, sending condolence cards on behalf of treating teams, and several members of the local treating team attending Riordan’s funeral. This was considered an important gesture of support to the family, coming together to honour and celebrate Riordan’s life, as per his wishes. The nurse practitioner and the psychologist also visited the family home, separately, in the weeks after Riordan’s death. Support included providing opportunities for the family to reflect on their experience of Riordan’s death and their feelings of loss. Approaches included active listening and support around expressed feelings of grief and loss and considering approaches to navigating the coming weeks and months. Information was also provided about community bereavement services they could access in coping with immediate and future impacts of loss.

## 4. Discussion

In this narrative case study, we describe the home-based care provided to one family as an example of holistic palliative care practice. The story told of Riordan’s care serves to honour experiences that were difficult and complex, and thus can be an important source of knowledge. We highlight the importance of care coordination and interprofessional collaboration in the planning, organisation, and delivery of care to meet the unique needs of the child and family. By exploring the experiences of the child and family, this report highlights the importance of comprehensive support, family involvement and open communication. The application of narrative inquiry not only enriches our understanding, but also provides actionable recommendations to enhance the provision of holistic and compassionate home-based paediatric palliative care (Box 1).

Box 1 **Recommendations for holistic and compassionate home-based paediatric palliative care.**

1. Introduce the care coordinator early, to foster trust and collaboration throughout the care journey.
2. Ensure the family knows who to call and when (e.g., for symptom management, at time of death).
3. Ensure that all aspects of care are completed with a patient and family-centred focus, respecting the unique cultural, emotional, and individual needs of each child and their family.
4. Encourage and support family conversations around care, seeking to clarify the child and siblings’ understanding, to enable wishes to be identified and included in goals for care.
5. Provide opportunities for separate child- and parent-focused reviews to allow children and parents to speak openly about their concerns without feeling the responsibility to protect their family from the impact of their disclosure.
6. Establish formal processes for interprofessional communication and collaboration.
7. Utilise formal documents to plan and manage symptoms, advance-care planning, and after-death care.
8. Include the family in care planning, to ensure cultural practices and rituals are understood and respected.
9. Address the emotional and psychological needs of the whole family by including/referral to appropriate allied health, pastoral-care, and support services.
10. Provide resources for memory-making and support families in their grief and bereavement process.
11. Where available, referral to hospice may be beneficial for respite, end-of-life or after-death care, or bereavement.
12. Develop clear protocols and guidelines for after-death care that include options for families to choose the location of care and post mortem tumour donation, if relevant.
13. Promote ongoing professional development for healthcare practitioners.
14. Establish mechanisms for regular peer support among healthcare practitioners.

Introducing early care coordination established a point of contact for the family, allowing for a therapeutic, trusting relationship to be developed. This helps to plan and prepare for goals of care and to anticipate the child and family needs. Effective interprofessional collaboration can facilitate seamless comprehensive home-based care. The use of case conferences across different institutions or physical locations assists with communication, ensuring all relevant professionals are aware of plans. In Riordan’s case, this collaboration was crucial in facilitating after-death care and post mortem tumour donation. Central to all palliative care provision is meticulous planning. The use of Symptom Management Plans, the advance-care planning documented in the Paediatric Acute Resuscitation Plan, and an After Death Care Plan, are all examples of formal documents that can support coordinated and effective home-based care [34].

It is crucial that families are included in developing these plans, so that cultural practices and rituals can be respected and included. Attention to the child and families’ need for emotional and psychological support can be facilitated by allied health professionals skilled in therapy, and/or the inclusion of appropriate pastoral care and support services. In Riordan’s case, the use of music therapy offered the whole family a space they could unite together in and create lasting memories. Addressing the emotional well-being of the child and family is integral to holistic paediatric palliative care. While the seminal work of Elisabeth Kübler Ross describes the emotional aspects of coping with death and dying, contemporary literature has evolved and expanded, recognizing that grief is complex and highly individualized [35]. In the case of a dying child, best practice recommendations recognise the parents as the experts in knowing their child. Health professionals play a critical role in supporting parents [36]. The decision of whether to involve a child in discussions about their care or to tell them they are dying are deeply sensitive issues. Such decisions should always be made with the child’ best interests in mind.

Palliative care practice is an emotionally complex and evocative process, and one which healthcare professionals report feeling unprepared for [37]. Several reasons exist for this, including receiving little formal education about palliative care, lack of confidence in the ability to manage patient and family care, and the practitioner’s own personal feelings of grief and loss [38]. It is important to acknowledge the effect of the inherent challenges and emotional toll of providing home-based palliative care on practitioners. In the case study of Riordan, there is learning for optimizing care for the child and family, and also for ensuring support for practitioners. Central to this are the roles of the interprofessional team and peer support, which are instrumental in ensuring practitioners do not feel professionally isolated [39]. Whether support is achieved through formal meetings, through supervision, or available in an ad hoc manner by telephone, practitioners providing home-based care need to have access to support. This is pivotal to maintaining their well-being and emotional resilience so they can provide holistic and compassionate care to families.

### Strengths and Limitations

We acknowledge the limitations of this case study, particularly the subjective description which limits generalizability. Subjective and judgmental elements are inherent aspects of narrative inquiry and case study descriptions. For the purposes of addressing our aim, this method provided the opportunity to describe in rich detail the home care provided to one family as an example of practice in action. In a society where death remains taboo, and healthcare practitioners frequently ask for greater support in caring for dying children and their families, this narrative may inform practice at both the individual and service-provider levels by providing a deeper sense of knowing [19]. Researchers and practitioners can benefit from a better understanding of the complexities involved in delivering palliative care in the home environment, as well as obtaining insights into how to coordinate the efforts of multiple service providers to ensure comprehensive support for the child and the family. Although the description is contextualised to our location and availability of resources, it can serve to inform other services striving to provide the best care possible in the home environment. When considered alongside other learning from empirical research in paediatric palliative care, the art and science of the complex decision making needed to provide high-quality care can be enhanced.

## 5. Conclusions

This report provides insights into the intricate landscape of home-based paediatric palliative care, describing the challenges and strategies involved in delivering holistic and compassionate care within the familiar environment of a child’s home. The narrative case study not only enriches our understanding but also offers actionable recommendations to enhance the provision of holistic and compassionate home-based paediatric palliative care. The nuances of care described offer an understanding of how, when, and why care was delivered, and may help other practitioners who provide care to children and families in similar contexts. The insights and recommendations outlined here can be used to optimize home-based paediatric palliative care programs, ultimately improving the quality of life for children and families facing life-limiting conditions.

## Data Availability

No new data were created or analysed in this study. Data sharing is not applicable to this article.
